# Zwitterionic Nanofibers of Super-Glue for Transparent and Biocompatible Multi-Purpose Coatings

**DOI:** 10.1038/srep14019

**Published:** 2015-09-11

**Authors:** Elisa Mele, José A. Heredia-Guerrero, Ilker S. Bayer, Gianni Ciofani, Giada G. Genchi, Luca Ceseracciu, Alexander Davis, Evie L. Papadopoulou, Markus J. Barthel, Lara Marini, Roberta Ruffilli, Athanassia Athanassiou

**Affiliations:** 1 Smart Materials, Istituto Italiano di Tecnologia (IIT), via Morego 30, 16163 Genoa, Italy; 2 Department of Materials, Loughborough University, Loughborough, Leicestershire, LE11 3TU, UK; 3 Center for Micro-BioRobotics @SSSA, Istituto Italiano di Tecnologia (IIT), viale Rinaldo Piaggio 34, 56025, Pontedera, Pisa, Italy; 4 Drug Discovery and Development Department, Istituto Italiano di Tecnologia, Via Morego 30, 16163 Genoa, Italy; 5 CEMES, CNRS, 29 rue J. Marvig, 31055 Toulouse Cedex 4, France

## Abstract

Here we show that macrozwitterions of poly(ethyl 2-cyanoacrylate), commonly called Super Glue, can easily assemble into long and well defined fibers by electrospinning. The resulting fibrous networks are thermally treated on glass in order to create transparent coatings whose superficial morphology recalls the organization of the initial electrospun mats. These textured coatings are characterized by low liquid adhesion and anti-staining performance. Furthermore, the low friction coefficient and excellent scratch resistance make them attractive as solid lubricants. The inherent texture of the coatings positively affects their biocompatibility. In fact, they are able to promote the proliferation and differentiation of myoblast stem cells. Optically-transparent and biocompatible coatings that simultaneously possess characteristics of low water contact angle hysteresis, low friction and mechanical robustness can find application in a wide range of technological sectors, from the construction and automotive industries to electronic and biomedical devices.

Cyanoacrylate monomers, commonly known as super glues or instant adhesives, are highly reactive and low viscosity liquids. They are widely used for repairing rubber, plastic and metallic parts in industrial and domestic applications, for sealing injured tissues in surgical and medical practices, as well as for fingerprint development in forensic laboratories^1^,^2^,^3^,^4^. They undergo polymerization within a few seconds in the presence of nucleophiles or traces of weak bases, for instance natural ambient humidity^1^, resulting in the formation of mechanically strong polymers with excellent adhesion properties, low toxicity and biocompatibility. Motivated by these distinguishing features, there is a growing interest in developing fabrication strategies that are able to structure cyanoacrylates in the form of nanofibers. In fact, nanofibrous mats have considerable impact on a broad range of sectors, including liquid filtration^5^,^6^, tissue regeneration^7^,^8^, biomedical devices^9^,^10^,^11^,^12^ and optoelectronics^13^,^14^.

However, the high reactivity and extremely rapid polymerization of cyanoacrylates, even upon contact with alcohols, water and slightly basic solvents, strongly limits their use for the production of one-dimensional (1D) nanostructures by physical methods, such as electrostatic spinning (ES). Hence, other strategies have been developed, such as vapor phase polymerization on finger print ridges^15^, co-electrospinning with other acrylic polymers^16^,^17^, or reaction with silane functionalized surfaces^18^. The main limitation of these methods, compared to the ES technique, is the difficulty to produce large amounts of pure and long cyanoacrylate fibers with well-defined morphology.

Herein, we show for the first time the electrospinning of highly uniform and long fibers of poly(ethyl 2-cyanoacrylate), PECA, without the assistance of any polymeric additive. In our approach, the zwitterionic polymerization of ethyl 2-cyanoacrylate (ECA) monomers is promoted by dimethyl sulfoxide (DMSO), leading to the formation of macrozwitterions of PECA that are easily electrospinnable. We demonstrate that the produced electrospun mats can be thermally transformed into transparent and textured coatings with unique wetting properties (hydrophilic and self-cleaning). Interestingly, the coatings behave as solid lubricants with low friction coefficient (0.019), excellent scratch resistance and durability. Furthermore, they are highly biocompatible as verified by the enhanced proliferation and differentiation of myoblast stem cells (C2C12). Finally, the proliferation of C2C12 can be spatially controlled by thermally treating only specific areas of the electrospun mat and consequently creating patterned PECA coatings. Direct electrospinning of cyanoacrylates is expected to open up new application areas associated with regenerative medicine and artificial implants, as well as with self-cleaning and lubricant polymer surfaces.

## Results

### Zwitterionic polymerization of PECA and nanofibers formation

**Figure 1:**
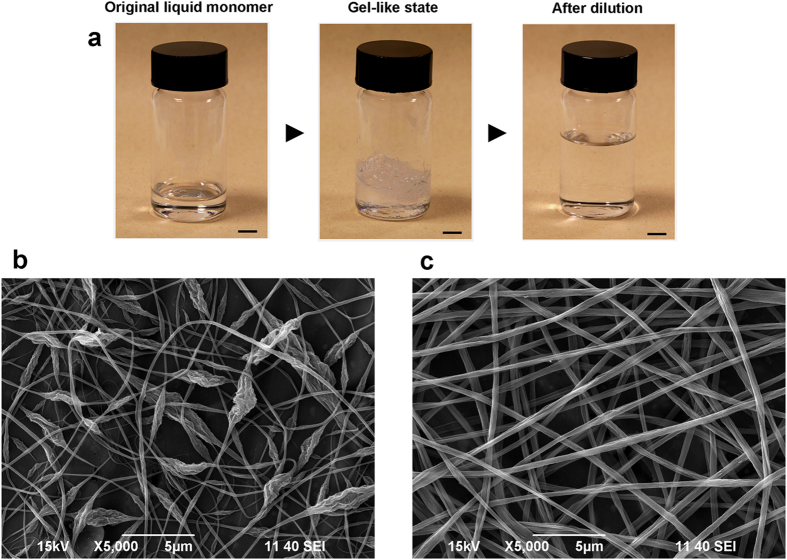
Zwitterionic PECA fibers. (**a**) Photographs of the different stages of preparation of the DMSO-modified ECA: from left to right, initial commercial liquid ECA, PECA gel after mixing ECA with DMSO, liquid solution of zwitterionic PECA in acetone. Scale bar = 0.5 cm. SEM images of the PECA fibers electrospun from acetone solutions at concentration of (**b**) 2.5% v/v and (**c**) 5.0% v/v.

In its initial formulation ECA is a transparent liquid with an average viscosity of 0.1 Pa·s (photograph on the left, Fig. 1a). Upon mixing it with DMSO at 1:1 volume ratio, an exothermic reaction took place with a consequent fast increment of the solution viscosity and the formation of a gelatinized system (photograph in the middle, Fig. 1a). This gel was thinned by acetone to obtain a crystal-clear solution (photograph on the right, Fig. 1a). In particular, DMSO-modified ECA solutions in acetone were prepared at concentrations of 2.5, 5.0 and 10.0% v/v for electrospinning. In contrast with the starting ethyl 2-cyanoacrylate, the polymer derived by reacting ECA with DMSO could be easily electrospun even with low applied voltages (5 kV). The morphology of the produced PECA fibers was investigated by scanning electron microscope (SEM). The fibers that were electrospun from DMSO-ECA acetone solutions at the lowest concentration (2.5% v/v) exhibited a non-uniform diameter throughout their length, since bead-like structures were present (Fig. 1b). In order to improve the size uniformity of the fibers, DMSO-ECA acetone solutions at a concentration of 5.0% v/v were prepared. In this way, networks of long and well-defined fibers having a diameter of (473 ± 58) nm were produced over large area, without the presence of beaded features (Fig. 1c). Fibers with bigger diameter were electrospun by further increasing the concentration of the DMSO-ECA polymer in acetone (10.0% v/v, Figure S1 in the Supplementary Information).

**Figure 2:**
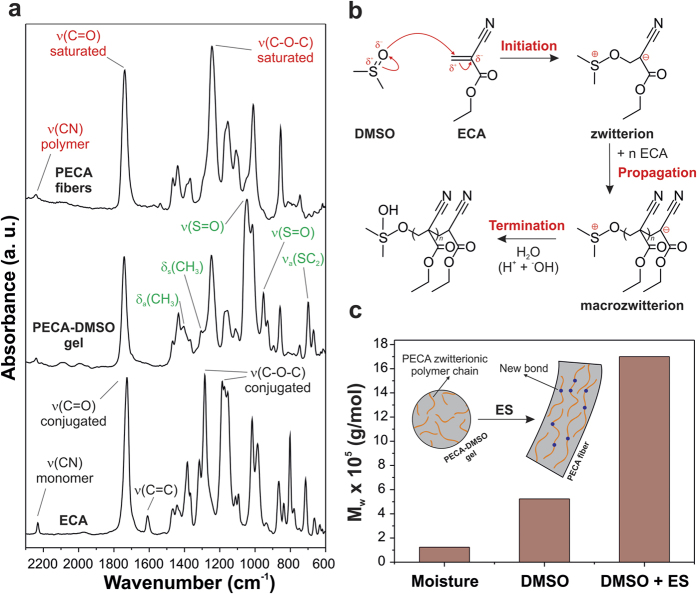
Chemical analysis of the produced fibrous mats. (**a**) From bottom to top, ATR-FTIR spectra of the ECA monomer, the DMSO-PECA gel and the electrospun zwitterionic fibers. The main bands associated with ECA (black color), PECA (red color) and DMSO (green color) are assigned. (**b**) Proposed scheme of the reactions occurring for the zwitterionic polymerization of ECA in DMSO (initiation, propagation and termination). For the termination reaction, the proton and hydroxide transfer from water molecules is shown. (**c**) Values of M_W_ for the cyanoacrylate polymerized under different conditions (moisture, DMSO and DMSO + ES). Inset: schematic representation of the combination of the zwitterionic chains during the ES process.

The reaction occurring between ECA and DMSO was studied by comparing the Fourier transform infrared spectroscopy (FTIR) spectra of the initial ECA monomers, the DMSO-modified ECA gel and the resulting electrospun fibers (Fig. 2a). Several bands assigned to the functional groups of the molecule of ethyl 2-cyanoacrylate can be easily identified in the spectrum of ECA: the C 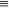 N stretching in monomer molecular environments at 2239 cm^−1^, the conjugated C = O stretching at 1732 cm^−1^, the C = C stretching at 1614 cm^−1^ and the conjugated C-O-C stretching modes at 1288 and 1188 cm^−1^ 1,19,20. On the other hand, the gel shows absorptions that can be ascribed both to DMSO (the CH_3_ asymmetrical bending at 1412 cm^−1^, the CH_3_ umbrella mode at 1308 cm^−1^, the S = O stretching modes at 1047 and 953 cm^−1^, and the SC_2_ asymmetric stretching at 698 cm^−1^)^21^ and to PECA (the C 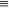 N stretching in polymer molecular environments at 2251 cm^−1^, the saturated C = O stretching at 1744 cm^−1^, and the single saturated C-O-C stretching at 1248 cm^−1^)^1^,^19^,^20^. Finally, the IR spectrum of the electrospun fibers consists of the typical vibrations of PECA without contributions of solvents (DMSO and acetone).

It has been demonstrated that polar solvents, like DMSO, can initiate the zwitterionic polymerization of *n*-alkyl 2-cyanoacrylates^22^. The oxygen of the sulfoxide functional group is a strong nucleophile (a hard Lewis base) and it can react with the unsubstituted *β*-carbon of the ECA monomer, which is a good electrophile center as consequence of the electronegativity of nitrile and ethyl ester groups. According to this model, we can state that the polymerization of the ECA monomers was initiated immediately after the mixing with DMSO and zwitterions were produced^23^,^24^. These zwitterions continued the polymerization (“propagation”) by reacting with the other monomers, forming macrozwitterions. The schematic representation of the process is reported in Fig. 2b.

We determined the mass-average molar mass (M_w_) of the resulting PECA macrozwitterions by Gel Permeation Chromatography (GPC) (Fig. 2c), obtaining a value of 5.2 × 10^5^ g mol^−1^ that was higher than the M_w_ of PECA polymerized by moisture (1.2 × 10^5^ g mol^−1^). In this last case, the polymerization was anionic and quickly terminated by the protons of the water molecules^25^,^26^, leading to shorter polymer chains. On the contrary, the termination of the zwitterionic polymerization by protons was disfavored in DMSO (the pK_a_ of methyl groups of dimethylsulfoxide is ∼35). The M_w_ of the electrospun polymer was the highest measured (17.0 × 10^5^ g mol^−1^) indicating the further combination of the macrozwitterions PECA chains in the fibers. We postulate that during the electrospinning process the charges located at the end groups of the PECA macrozwitterions were affected by the applied electric field and the intermolecular association of the opposite charges of two polymeric zwitterions was promoted (inset in Fig. 2c). The polymerization reaction was terminated through the formation of the fibers, probably as a consequence of the complete evaporation of DMSO and of proton and hydroxide transfer from water molecules of the environment^22^,^25^,^27^,^28^.

### Hydrophilic textured PECA coatings with low liquid adhesion and anti-staining performances

**Figure 3:**
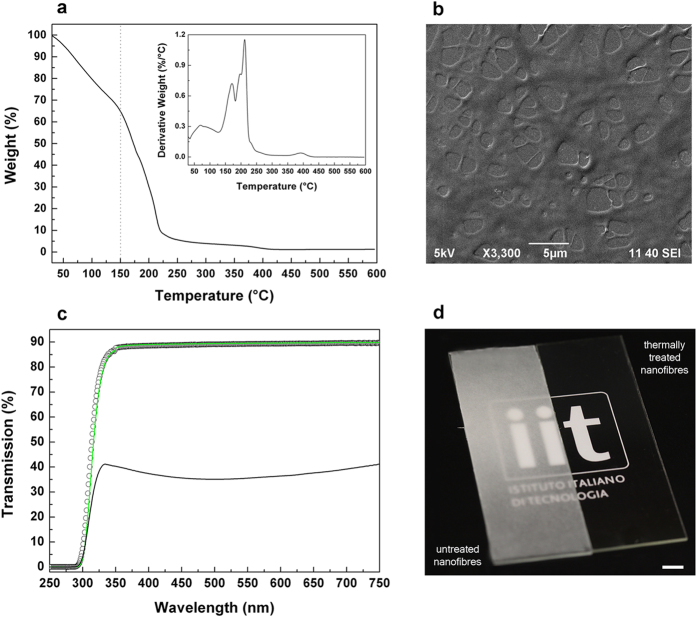
Transparent coatings from thermally-treated PECA fibers. (**a**) TGA thermogram and relative derivative curve (inset) of the PECA fibers. (**b**) SEM image of the textured PECA coating produced by thermally treating the electrospun fibers at 150 °C. (**c**) Optical transmission spectra of the glass substrate (white circles) and of the electrospun mat deposited on the glass substrate before (solid black curve) and after (solid green curve) the thermal treatment. (**d**) Photograph of the as-prepared PECA fibers on the glass substrate (sample on the left) and of the textured thermally-treated coating on the glass substrate (sample on the right). Scale bar = 0.5 cm. Use of the institutional logo with permission of Istituto Italiano di Tecnologia.

The produced PECA fibers were used for the creation of textured coatings with special wetting properties. Initially, glass substrates were uniformly coated with layers of PECA nanofibers and then thermally treated at 150 °C for 20 s on a hot plate. The temperature for the treatment of the fibrous networks was selected in order to obtain coatings with a high degree of optical transparency, while limiting the thermal degradation of the polymer. TGA measurements in Fig. 3a indicate that the PECA electrospun mats had the maximum degradation at 210 °C, and they completely degraded at 300 °C, in agreement with previous reports on PECA^29^,^30^. We measured a weight loss of 35% at 150 °C, mainly due to the complete evaporation of water that was trapped inside the porosity of the electrospun web (at a temperature lower than 100 °C) and only partially due to the thermal degradation of the PECA fibers. Real-time observations of the dynamics of the formation of the textured PECA coatings (Figure S2 in the Supplementary Information) show, as expected, that the fusion of the fibers started from the layers closer to the substrate, which were in direct contact with the heating source. Then, it propagated through the upper layers of fibers. As result, the fibers fused together, forming interconnected features that recalled the entangled geometry of the initial fibrous network (Fig. 3b). It is worth noting that the produced micro-structured coatings were highly transparent to visible light, with a transmission spectrum (solid green line in Fig. 3c) comparable to that of the pristine glass substrate (white circles in Fig. 3c). In fact, the thickness (lower than 100 nm) and the low surface roughness of the coatings guaranteed a reduced scattering of the light, leading to high transparency^31^. On the contrary the glass slide coated with the PECA fibers, before the thermal treatment, was optically opaque (solid black line in Fig. 3c). A direct comparison between the as-prepared (sample on the left) and treated (sample on the right) fibrous network is shown in Fig. 3d.

The textured coatings were analyzed by Raman spectroscopy, in order to study possible changes of the chemical composition of PECA after the heating at 150 °C (Figure S3 of the Supplementary Information). The measurements indicated that even after the thermal treatment the major characteristic vibrations of the cyanoacrylate polymer were retained. Therefore, the heating process induced modifications on the morphology and physical organization of the PECA fibers without affecting their chemical composition and vibrational spectroscopic signature.

**Figure 4:**
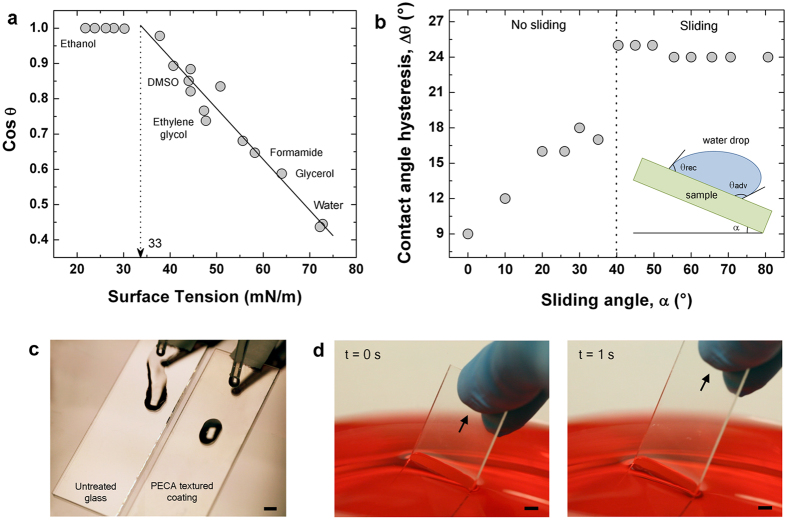
Anti-staining performances of the PECA textured coatings. (**a**) Experimental data (grey circles) of the cosine of the contact angle as function of the surface tension of the analyzed liquids. The solid line is the best linear fit of the data. (**b**) Experimental data (grey circles) of the contact angle hysteresis as function of the sliding angle. (**c**) Photograph of drops of water impacting and sliding on two glass substrates: one coated with the thermally treated PECA fibers (sample on the right) and the other one uncoated (sample on the left). Scale bar = 0.5 cm. (**d**) Temporal sequence showing a double-side coated glass substrate pulled out from a water bath. The water was colored in red for visualization purposes. Scale bar = 0.5 cm.

One interesting characteristic of the produced PECA coatings is their self-cleaning ability. The solid surface energy of the coatings was estimated using different liquids (tricresyl phosphate, DMSO, diethylene glycol, bromonaphtaline, ethylene glycol, diiodomethane, formamide, glycerol) and mixtures of water and ethanol (further details in Table S1 of the Supplementary Information). The plot in Fig. 4a shows the value of the cosine of the measured contact angle (*cos θ*) as a function of the surface tension of the liquid used. For the cases of incomplete wetting (liquids with finite contact angles), the data were fitted with a line. Its intercept at *cos θ* = 1 is regarded as the estimated solid surface energy for the PECA coatings, which was equal to 33 mN/m. Indeed, this value indicates a hydrophilic surface with a water contact angle of about 65°. Interestingly, the water droplets started sliding once the substrates were tilted to 40^°^ (sliding angle *α*) with a constant contact angle hysteresis (Δ*θ*) of approximately 24°, as shown in Fig. 4b. In fact, when the water drops impacted on the coating (sample on the right in Fig. 4c) they slid away without leaving any trace of liquid behind them. On the contrary, as expected, the uncoated glass substrate was easily wet by water (sample on the left in Fig. 4c). A further demonstration of the anti-staining performance of the PECA textured coatings is shown in Fig. 4d, where a glass substrate with both sides coated was dipped in a bath of water (colored red). When the sample was pulled away from the water, both surfaces appeared completely dry.

### Low friction coefficient and mechanical robustness of the PECA coatings

**Figure 5:**
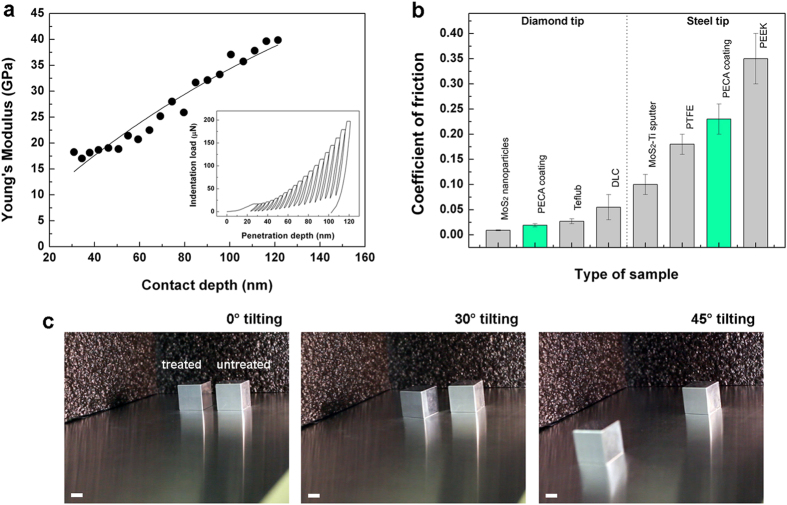
Hardness and friction coefficient of the PECA coatings. (**a**) Experimental data (black circles) of the Young’s modulus of the PECA textured coating as a function of the contact depth. The solid line is the best fit with Mencik’s equation. Inset: typical continuous multi-cycle curve of indentation load vs. the penetration depth for the studied coatings. The Young’s modulus was calculated from each loading-unloading cycle. (**b**) Friction coefficient of the PECA coating (in green) measured by diamond and steel tip. Reference materials (in grey) were used for comparison. (**c**) Temporal sequence of the sliding of two stainless steel cubes at different tilting angles: one coated with PECA (cube on the left) and one uncoated (cube on the right). Scale bar = 1 cm.

Additional properties that the textured PECA coatings possess are mechanical hardness, low friction coefficient and high durability, as demonstrated by mechanical analysis. Curves of indentation load versus penetration depth (inset in Fig. 5a) were recorded and used to calculate the Young’s modulus of the coatings. In Fig. 5a, the values of the elastic modulus (*E*_*c*_) are shown as a function of the contact depth (*h*_*c*_, calculated according to the Oliver and Pharr method)^32^. The experimental data were fitted with the following exponential law 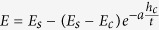
33, where the *E*_*s*_ is the Young’s modulus of the glass substrate (72 GPa), *a* is a fitting constant and *t* is the film thickness. The fitting of the data at “zero penetration” (solid line in Fig. 5a) yielded a value of the modulus of (3.1 ± 0.5) GPa that is consistent with the results reported for other acrylic polymers^34^. Moreover, the hardness of the PECA coating was calculated as the contact area at maximum load^32^. Quasi-static indentations were performed at three maximum loads (20, 30 and 50 μN) with loading and unloading times of 30 and 15 s, respectively. The measurements were conducted at a depth of about one third of the film thickness (Figure S4 in the Supplementary Information), which is a method generally accepted for studying the hardness of soft coatings on stiff substrates, as in our case^35^. In fact, in this way the effect of the substrate underneath is negligible^36^. A value of hardness equal to (0.44 ± 0.13) GPa was calculated, which is comparable to that one of other hard polymer coatings^37^,^38^,^39^. Furthermore, the coefficient of friction (CoF) of the PECA thin films was measured by a diamond tip, obtaining a value of 0.019 ± 0.003. We compared this result with the performances of products conventionally used and accepted as dry lubricants (Fig. 5b): CRC Teflub (a Polytetrafluoroethylene-based hydrophobic compound), Diamond-like carbon (DLC, an amorphous carbon material with high wear resistance) and thin films of MoS_2_ nanoparticles. We observed that the PECA coatings exhibited a CoF close to that one of films of MoS_2_ nanoparticles (range 0.008–0.01)^40^ and CRC Teflub (value 0.027 ± 0.005), and even lower than DLC (range 0.03–0.08)^41^. Moreover, analyses that were conducted by a stainless steel tip (Fig. 5b) underlined that the friction coefficient of the PECA textured coatings (0.23 ± 0.03) was lower than most of the polymer films proposed as solid lubricants^42^,^43^, such as Polyether ether ketone (PEEK, in the range of 0.3–0.4)^44^, and comparable to Polytetrafluoroethylene (PTFE, in the range of 0.16–0.20)^45^. As expected, the friction coefficients measured by the diamond tip were lower than those obtained using the steel tip, due to the intrinsic properties of diamond and the higher Hertzian pressure^46^,^47^. We can state that the thin films produced by the thermal treatment of the electrospun PECA fibers performed as a solid lubricant, reducing the friction between two sliding contact interfaces. For visualization purposes, we monitored the sliding of two stainless steel cubes (side of 2 cm) on a steel surface: one having the contact surface covered with the PECA coating (sample on the left) and the other one untreated (sample on the right). As visible in Fig. 5c, the treated cube started sliding at a tilting angle of 30°, differently from the uncoated one that slid at an angle higher than 55°.

Desirable properties for a solid lubricant are scratch resistance and durability. Indentation analyses allowed us to evaluate the scratch resistance of the produced PECA coatings. Upon a pressure of 1 GPa, generated on the coating by a diamond tip, low deformation and lack of appreciable delamination events were recorded (Figure S5 of the Supplementary Information). A permanent deformation was observed only at higher critical load (2.8 ± 0.8 N that corresponds to a Hertzian pressure of 1.4 GPa). It is most likely that the thermal treatment improved the adhesion of the PECA coating on the substrate, with consequent effect on its mechanical stability.

### Analysis of the proliferation and differentiation of myoblast cells on the textured coatings

**Figure 6:**
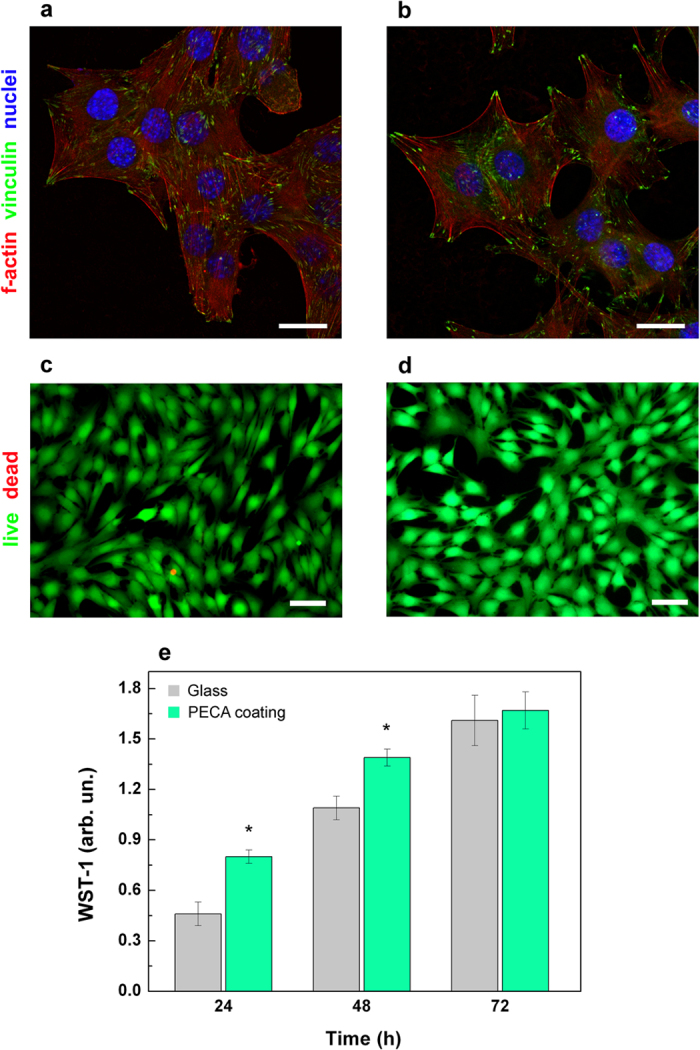
Adhesion and proliferation of C2C12 myoblasts. Confocal images of focal adhesions of C2C12 myoblasts cultured on (**a**) the glass substrate and on (**b**) the PECA textured coating at 24 h from seeding. F-actin, vinculin and nuclei were stained in red, green and blue, respectively. Scale bar = 20 μm. Live/Dead^®^ (green/red) staining of myoblasts shows comparable cell viability on (**c**) glass and on (**d**) the cyanoacrylate sample at 24 h from seeding. Scale bar = 50 μm. (**e**) WST-1 assay of the metabolic activity of myoblasts within 48 h from seeding on glass (gray) and on the PECA textured coating (green). *p < 0.05.

The properties of the PECA coatings, described until here, make them excellent candidates for a vast range of applications, such as optoelectronics, automotive, and tissue engineering. For this latter case, one important requirement is the biocompatibility of the coating. In order to assess this point, studies were performed with the C2C12 skeletal myoblast line that was selected for the capability of proliferation and differentiation. Glass substrates were used as control. The adhesion of C2C12 myoblasts on the glass substrate (Fig. 6a) and on the PECA coating (Fig. 6b) was analyzed by confocal microscopy after 24 h of culture. Cell spreading, cytoskeletal conformation (f-actin stained in red) and expression of vinculin (stained in green) were similar for both samples, with focal adhesions that followed typical patterns at the edges of the cell bodies. These observations indicate that the myoblasts well adhered and spread on the textured PECA coatings like on the glass substrate, as also confirmed by the SEM imaging (Figure S6). Furthermore, the cell viability was comparable for the two substrates after 3 days of culture, as demonstrated by the live/dead staining of proliferating myoblasts (Fig. 6c,d; in green viable cells, in red necrotic cells). Interestingly, the metabolic activity, assessed by WST-1 assay, significantly increased on the textured PECA coatings within 48 h from seeding (Fig. 6e), suggesting that the topographical cues were able to elicit an improved metabolic response in the early stages of cells/material interactions. In fact, surface texturing is nowadays recognized as an essential requisite for favoring the integration of materials with biological environments^48^,^49^.

**Figure 7:**
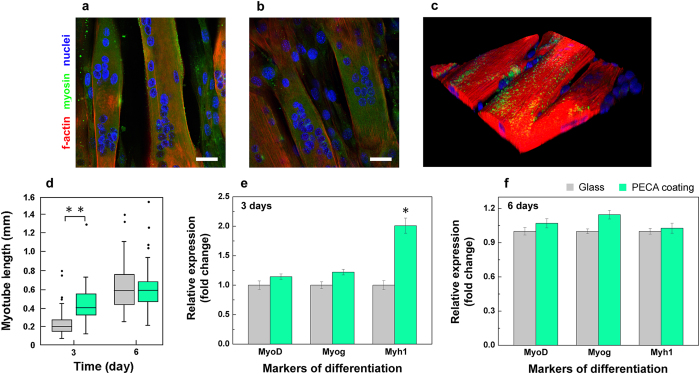
*In-situ* differentiation of C2C12 in myotubes. Confocal microscopy images of the C2C12 myoblasts fuse in myosin-positive myotubes on (**a**) the glass substrate and on (**b**) the PECA coating after 72 h of differentiation. F-actin, myosin and nuclei were stained in red, green and blue, respectively. Scale bar = 20 μm. (**c**) 3D rendering of myotubes on the cyanoacrylate sample from confocal images of cultures after 72 h of differentiation. Volume: 210 × 210 × 25 μm^3^. (**d**) Myotube lengths measured from confocal images after 3 days of differentiation. **p < 0.01. Transcription of *MyoD*, *Myog* and *Myh1* quantified with qRT- PCR after (**e**) 3 and (f) 6 days of differentiation. *p < 0.05. Glass substrate in gray and PECA coating in green.

The high biocompatibility of the PECA coatings is also demonstrated by the differentiation *in-situ* of the C2C12 myoblasts. Indeed, confocal images of the differentiating cell cultures showed that the myoblasts fused in myosin-positive myotubes after 6 days of differentiation (Fig. 7a,b), as also observed by SEM imaging (Figure S7 in the Supplementary Information). As a representative example, 3D rendering of a section of the differentiated cultures with multinucleate myotubes is reported in Fig. 7c. A quantitative analysis of the cell differentiation in terms of myotube length further confirmed an improved response of the cells in the first days of cultures. In particular, the myotubes developed on the PECA coating were significantly longer (about 90%, p < 0.01) than those on standard glass substrates (Fig. 7d). Furthermore, the enhanced differentiation was confirmed at a gene level by quantitative RT-PCR analysis. High up-regulation of *Myh1* (about 2-fold, p < 0.05), a late marker of muscle differentiation, was observed for the cell cultures after 3 days of differentiation onto the textured coatings. The transcription of the early markers of differentiation (*MyoD* and *Myog*) instead appeared unaffected after both 3 and 6 days of differentiation (Fig. 7e,f).

**Figure 8:**
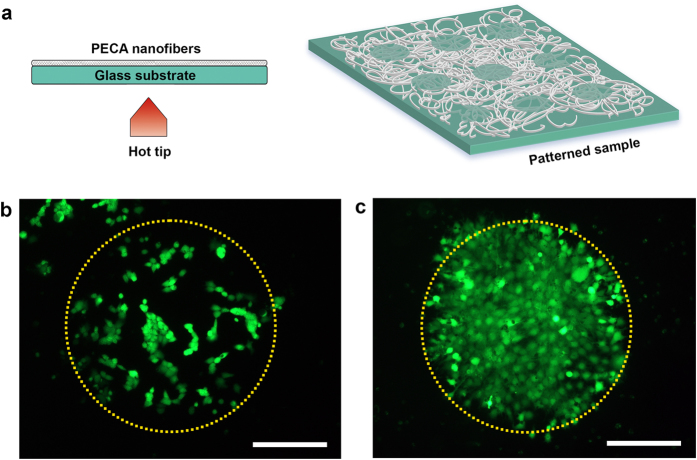
Patterned fibrous mats for spatially-controlled cell growth. (**a**) Schematic representation of the set-up used for the production of the PECA samples patterned with circular spots. Confocal images of (**b**) the spatial confinement of the cells (live cells in green) at 24 h from seeding and of (**c**) the proliferating cells after 72 h of culture. The dotted yellow circles are guides for the eye. Scale bar = 500 μm.

Finally, spatial control over the proliferation of the myoblasts was achieved by producing patterned PECA coatings through the thermal treatment of only specific areas of the electrospun mat. A hot metallic tip was brought in contact with the surface of the glass substrate that was not covered with the electrospun mat. In this way, patterns consisting of circular transparent spots surrounded by the network of PECA fibres were created (Fig. 8a). As shown in Fig. 8b, the obtained samples were able to promote the spatial confinement of the cells as early as 24 h from seeding. The localized cell growth could be maintained during the proliferation of the myoblasts, as demonstrated by the formation of cell populations with perfectly circular shape (Fig. 8c; cells stained in green with calcein) after 72 h of culture. All of the collected evidences support the use of PECA structured coatings for biological applications, given their optimal biocompatibility and ability to sustain cellular functions. Moreover, the possibility of easy patterning allows the spatial control of the cellular adhesion and proliferation, with important implications for tissue engineering^50^,^51^.

## Discussion

Previous attempts to fabricate cyanoacrylate fibers by the electrospinning technique have required both the use of poly(methyl methacrylate) as additive and specific modifications of the experimental procedure^16^,^17^, having to face the rapid moisture-driven cross-linking of the super-glue. The approach that we have developed, on the contrary, allows carrying out the ES process in the conventional way, starting from acetone solutions of DMSO-PECA. In fact, DMSO initiates the polymerization of ECA and promotes the formation of a gel mainly constituted by PECA macrozwitterions. These latter self-assemble in long and well-defined fibers when they are electrospun. Therefore, pure PECA fibers can be easily produced.

TGA analysis and Raman spectroscopy of the PECA mats reveals that they can be processed at 150 °C without chemical degradation of the polymer but with changes only in the physical structure of the fibrous network. Indeed, when a dense network of PECA fibers is deposited onto a glass substrate and thermally treated, textured and optically transparent coatings were created. The investigation of the wetting behavior of the coatings reveals their self-cleaning and anti-staining ability, even if they are hydrophilic. Moreover, differently from previously reported transparent films with liquid repellency^52^,^53^,^54^, indentation and scratch resistance tests point out that the PECA coatings perform as dry lubricants, thanks to the low friction coefficient (0.019 with a diamond tip), hardness and durability. Besides possessing these properties, the PECA textured films are also highly biocompatible, as confirmed by studies conducted on C2C12 skeletal myoblasts. The cells are not only able to well adhere and proliferate on the coatings, with an enhanced metabolic activity, but also to differentiate in significantly long myotubes. Finally, patterns of treated and untreated regions can be easily created on the electrospun PECA mat in order to spatially control and localize the growth of the cells.

We envisage that the sector that more than others could benefit from all properties of the here presented PECA coatings is that one of biomedical devices. Protective layers with low friction coefficient and high biocompatibility are particularly required for the treatment of hip and knee joints, coronary stents, and heart valves^55^.

## Methods

### Electrospinning procedure

Equal volumes of low viscosity ECA super glue (Permabond 105, Sigma Aldrich) and reagent grade DMSO (Sigma Aldrich) were mixed in a glass vial. The mixture reached a gel-like consistency within 1 minute, and remained stable in this form for weeks under ambient conditions. Solutions for ES were prepared by diluting the zwitterionic PECA gel with acetone to obtain a concentration varying from 2.5 to 10.0% by volume. A plastic syringe with a stainless-steel 23-gauge needle was filled with the acetone solution of PECA and connected to a syringe pump (NE-1000, New Era Pump Systems, Inc.) working at a flow rate of 3 mL h^−1^. The solution was electrospun at a voltage of 5 kV, controlled by a high-voltage power supply (EH40R2.5, Glassman High Voltage, Inc.). The PECA fibers were collected on an aluminum plate placed at a distance of 15 cm from the needle.

### Fabrication of textured cyanoacrylate coatings

Uniform layers of PECA nanofibers were deposited onto glass substrates that were fixed on the aluminum collector, and then thermally treated on a hotplate at 150 °C for 20 seconds. After the treatment, transparent textured coatings were obtained. The thickness of these coatings was controlled by acting on the duration of the electrospinning procedure. PECA coatings having a thickness ranging from 30 to 100 nm were produced by varying the ES time from 15 to 60 minutes, respectively. Furthermore, patterns of circular spots (diameter of 1 mm) were created by a metallic sharp tip that was heated at a temperature of 150 °C. The tip was placed in contact with the glass substrate, previously coated with the PECA fibers for 5 seconds, allowing the local thermal treatment of the electrospun mat and the formation of transparent circular areas.

### Chemical characterization

The molar mass of the original ECA product, the PECA gel and the electrospun polymer was determined by Gel Permeation Chromatography (GPC). The measurements were carried out on an Agilent 1260 Infinity quaternary LC system consisting of an Agilent 1260 Infinity quaternary pump (G1311B), autosampler (G1329B), two PLGel 5 μm MIXED-C columns (kept at 25 °C) and refractive index detector (G1362A). Tetrahydrofuran (THF) was used as eluent using a flow rate of 1 mL min^−1^. The system was calibrated with an Agilent PMMA Calibration Kit M-M-10 (M_n_ = 875–1,677,000 g mol^−1^).

Infrared analysis was performed using an Attenuated Total Reflectance (ATR) accessory (MIRacle ATR, PIKE Technologies) coupled to a Fourier Transform Infrared (FTIR) spectrometer (Equinox 70 FT-IR, Bruker). All spectra were recorded in the range from 4000 to 600 cm^−1^ with a resolution of 4 cm^−1^, accumulating 128 scans. In a typical measurement, the sample was gently collocated on the spot of ATR accessory and slowly pressed. In order to ensure the reproducibility of the data, three samples of each type were measured. Raman spectra were collected at ambient conditions using a Horiba Jobin Yvon LabRAM HR800 μRaman spectrometer, equipped with a microscope. A 632.8 nm excitation line, in backscattering geometry through a 50X objective lens, was used to excite the specimens, at low power of ~100 mW. The experimental set-up consists of a grating 600 lines/mm with spectral resolution of approximately 1 cm^−1^.

### Morphological analysis

The morphology of the produced PECA nanofibers and coatings was characterized by scanning electron microscopy (SEM), using a JEOL JSM-6490LA microscope working in high-vacuum mode, with an acceleration voltage of 15 kV. Videos of the formation of the textured coatings were recorded by a high speed camera IDT MotionXtra NX4S2, working at a maximum frame rate of 1000 Hz. Finally, photographs of the fibrous mats and coatings were taken by a Canon EOS 5D (macro lens 100 mm).

### Thermal and mechanical characterization

The thermal degradation behavior of the electrospun nanofibers was investigated by thermo-gravimetric analysis (TGA) using a Q500 analyzer from TA Instruments. The measurements were performed under inert N_2_ atmosphere with a flow rate of 50 mL/min in a temperature range from 30 to 600 °C at heating rate of 10 °C/min.

The elastic modulus and the hardness of the PECA textured coatings were evaluated by nanoindentation using a CSM Instruments Ultra Nanoindentation Tester (UNHT). Samples with a thickness of (200 ± 40) nm, measured with a profilometer, were prepared for reducing the effect of the substrate. Indentation measurements were conducted by loading-unloading cycles with incremental maximum load for obtaining values of Young’s modulus as a function of the penetration depth. Each test consisted of 20 cycles, increasing the maximum load from 20 to 200 μN according to a quadratic law. The loading and unloading times were kept constant at 20 s and 10 s, respectively. A pause of 20 s was held at the maximum load in order to allow the saturation of the viscous deformation.

The tribological properties of the PECA coatings were investigated by a CSM Micro Combi scratch tester. Scratches having a length of 2 mm were produced by two different types of tips: a 0.1 mm-radius spheroconical diamond tip and a 0.5 mm-radius stainless steel sphere. The tips were moved laterally with a rate of 1 mm min^−1^ while applying a normal load (*F*_*n*_) ranging from 0.2 to 1 N. The average contact pressure during the tests was calculated through a Hertzian model. The lateral force (*F*_*l*_) was recorded and the coefficient of friction (*μ*) was calculated as *μ* = *F*_*l*_*/F*_*n*_. Additionally, scratch resistance measurements were conducted using a diamond tip, applying normal load that increases up to 5 N. The adhesion of the coatings to the glass substrates was estimated by optical microscopy, whereas the resistance to permanent deformation was evaluated through measurements of the residual depth after scratching.

### Wettability studies

The wetting properties of the PECA textured surfaces were characterized by measuring static liquid contact angles as well as water contact angle hysteresis during substrate tilting experiments. Sessile droplets (volume ∼10 μL) attached to the tip of the needle were gently lowered to the target surface to form an equilibrium contact angle. The measurements were conducted using a standard contact angle goniometer equipped with a charge-coupled device (CCD) camera and image processing software. The average values of 10 measurements were reported. A surface energy estimation method, known as Saito method, was used to quantify the hydrophobicity of the textured PECA coatings after thermal treatment^56^. According with this method, ten different probe liquids were used to estimate the surface energy of the coating along with the polar and non-polar (dispersive) components.

The Saito method is a modified geometric approach for the estimation of the mean surface energy^56^. For brevity, only the equation that can be used to estimate surface energy is shown here:


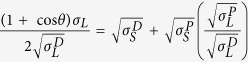


where *θ* stands for the static liquid contact angle, σ_*L*_, 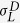 and 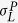 stand for liquid surface tension, dispersive component of the liquid surface tension, and the polar component of the liquid surface tension, respectively. For the solid surface, 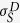 and 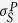 are the dispersive and polar components of the solid surface energy. Plotting 
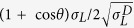
 versus 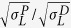 for various probe liquids enables the estimation of the dispersive and polar components of the solid surface energy. The slope and the intercept yield the polar and dispersive component, respectively.

### Biocompatibility assays

C2C12 mouse myoblasts (CRL-1772 ATCC) were seeded at a density of 5000 cells/cm^2^ on the thermally treated cyanoacrylate substrates. Bare glass coverslips were considered as control substrates. Cells were cultured under proliferative medium, prepared with Dulbecco’s modified Eagle’s medium (DMEM), 10% fetal bovine serum (FBS), 100 IU/mL penicillin, 100 μg/mL streptomycin and 2 mM L-glutamine (all reagents were from Life Technologies). Normal culture conditions (37 °C and 5% CO_2_ saturated humidity atmosphere) were applied, and culture medium was changed every three days. Differentiation of C2C12 myoblasts in myotubes was induced by supplying 80–90% confluent cultures with differentiative medium, prepared with DMEM, 1% FBS, 1% Insulin-Transferrin-Sodium Selenite (ITS, Sigma), 100 IU/mL penicillin, 100 μg/mL streptomycin, and 2 mM L-glutamine. Differentiative medium was changed every day. Biocompatibility of the substrates was evaluated through WST-1 assay (2-(4-iodophenyl)-3-(4-nitophenyl)-5-(2,4-disulfophenyl)-2 H-tetrazolium monosodium salt, BioVision). The assay was performed at 24, 48, and 72 h from cell seeding by incubating cultures with a 1:11 dilution of WST-1 reagent in proliferative medium for 2 h. Absorbance of the supernatants was read at 450 nm with a microplate reader (Victor3, Perkin Elmer).

Viability was further investigated with the Live/Dead® viability/cytotoxicity Kit (Molecular Probes). At 72 h from cell seeding, the cultures were incubated with 2 μM calcein AM and 4 μM EthD-1 in phosphate buffer saline (PBS) at 37 °C for 10 min, and then observed with an inverted fluorescence microscope (TE2000U, Nikon) equipped with a cooled CCD camera (DS-5MC USB2, Nikon) and with NIS Elements imaging software. Cell interactions with the substrates were investigated on both proliferating and differentiating cultures by immunocytochemistry staining of vinculin, a protein involved in focal adhesions, and of myosin heavy chain, a typical marker of muscle cell differentiation. The samples were first fixed in 4% paraformaldehyde (PFA) in PBS for 20 min, and then incubated with 1 mg/mL sodium borohydride in PBS for 10 min to reduce aspecific fluorescence and autofluorescence. Cell membranes were permeabilized with 0.1% Triton X-100 in PBS for 15 min. Antibody unspecific binding sites were saturated with 10% goat serum in PBS for 1 h, and, subsequently, a primary antibody (IgG anti-vinculin antibody, Millipore, 1:75 diluted in 10% goat serum or polyclonal IgG anti-myosin, Sigma, 1:100 diluted in 10% goat serum) was added. After 30 min of incubation at 37 °C, the samples were rinsed with 10% goat serum. Then, a secondary green fluorescent antibody (Invitrogen) diluted 1:250 in 10% goat serum was supplied with 100 μM TRITC-phalloidin (Sigma) for f-actin staining, and 1 μM DAPI for nucleus counterstaining. After 30 min of incubation at 25 °C, the samples were rinsed with 0.45 M NaCl in PBS for 1 min to remove weakly bound antibodies, and observed with a confocal laser scanning microscope (C2s, Nikon). The interaction between muscle cells and substrates was further investigated through SEM imaging of both proliferating (after 3 days) and differentiating (after 6 days) cultures. Cells were fixed with two sequential incubations with 4% PFA (at 4 °C for 30 min) and with a 2.5% glutaraldehyde in PBS (at 4 °C for 2 h). Samples were then dehydrated through an ethanol gradient (0, 25, 50, 75 and 100%), overnight dried, and gold-sputtered in a Quorum sputter coater before SEM observation through a Dual-Beam system (FEI Helios 600).

The transcription of muscle differentiation marker genes (MyoD, *MyoD*; myogenin, *Myog*; myosin heavy chain, *Myh1*) was evaluated with quantitative real-time RT-PCR (qRT-PCR). Total RNA was isolated by using High Pure RNA Isolation kit (Roche) following the manufacturer’s protocol. Retro-transcription of 400 ng of RNA into cDNA was performed with 4 μl of iScriptTM Reverse Transcription Supermix (Bio-Rad) in a total volume of 20 μL. The samples were incubated at 25 °C for 5 min, at 42 °C for 45 min, and then at 48 °C for 15 min. The enzyme was inactivated at 85 °C for 5 min, and finally the sample volume was increased 10 times with ultrapure water (MilliQ, Millipore). Quantitative RT-PCR was performed with a CFX Connect™ Real-Time PCR Detection System (Bio-Rad). Results were normalised to the transcription levels of a selected reference gene, glyceraldehyde 3-phosphate dehydrogenase (*Gapdh*). The obtained cDNA (5 μL) was mixed with 1 μL of specific forward and reverse primers (8 μM), 4 μL of ultrapure water, and 10 μL of SsoAdvancedTM SYBR®Green Supermix (Bio-Rad). The thermal protocol was applied with one cycle of 30 s at 98 °C and 40 cycles at 98 °C for 3 s and 60 °C for 7 s. At the end of amplification, a temperature ramp from 65 °C to 95 °C with 0.5 °C/s increments was performed to exclude unspecific products through melting curve results analysis. Each assay included “no template” sample and all tests were carried out in triplicate. The cycle threshold (*Ct*) value relative of control sample was adopted as reference for the calculation of ΔΔ*Ct* (difference between Δ*Ct* values, deriving from difference between *Ct* of target and reference gene) for the other samples. Primer sequences (forward and reverse) of the investigated genes are reported in Table S2 (Supplementary Information).

## Additional Information

**How to cite this article**: Mele, E. *et al.* Zwitterionic Nanofibers of Super-Glue for Transparent and Biocompatible Multi-Purpose Coatings. *Sci. Rep.* **5**, 14019; doi: 10.1038/srep14019 (2015).

## Supplementary Material

Supplementary Information
