# Supplementary material for: Zwitterionic Nanofibers of Super-Glue for Transparent and Biocompatible Multi-Purpose Coatings
Source: Sci Rep. 2015 Sep 11;5:14019. doi: 10.1038/srep14019 (PMC4566136; doi:10.1038/srep14019)
Supplement: Supplementary Information [file srep14019-s1.pdf]

Supplementary Information

Zwitterionic Nanofibers of Super-Glue for Transparent and Biocompatible Multi-Purpose Coatings

Elisa Mele,[1,2,*] José A. Heredia-Guerrero,[1] Ilker S. Bayer,[1] Gianni Ciofani,[3] Giada G. Genchi,[3] Luca Ceseracciu,[1] Alexander Davis,[1] Evie L. Papadopoulou,[1] Markus J. Barthel,[4] Lara Marini,[1] Roberta Ruffilli,[5] Athanassia Athanassiou[1,*]

[1] Smart Materials, Istituto Italiano di Tecnologia (IIT), via Morego 30, 16163 Genoa, Italy.

[2] Department of Materials, Loughborough University, Loughborough, Leicestershire, LE11 3TU, UK.

[3] Center for Micro-BioRobotics @SSSA, Istituto Italiano di Tecnologia (IIT), viale Rinaldo Piaggio 34, 56025, Pontedera, Pisa, Italy.

[4] Drug Discovery and Development Department, Istituto Italiano di Tecnologia, Via Morego 30, 16163 Genoa, Italy.

[5] CEMES, CNRS, 29 rue J. Marvig, 31055 Toulouse Cedex, France.

E-mail addresses: e.mele2@lboro.ac.uk; athanassia.athanassiou@iit.it

Table S1: Values of surface tension and contact angle for the different liquids.

**Probe liquid**	**Surface tension (mN/m)**	**Contact Angle (º)**
Water	72.8	63.6
Glycerol	64.0	54.0
Formamide	58.2	49.7
5% v/v Ethanol/Water	55.6	47.1
Diiodomethane	50.8	33.4
Ethylene glycol	47.7	42.5
10% v/v Ethanol/Water	47.3	40.0
Bromonaphtaline	44.4	27.9
Diethylene glycol	44.4	34.8
DMSO	44.0	31.7
Tricresyl phosphate	40.7	26.7
20% v/v Ethanol/Water	37.8	12.0
40% v/v Ethanol/Water	30.2	3.0
50% v/v Ethanol/Water	27.9	0
60% v/v Ethanol/Water	26.2	0
80% v/v Ethanol/Water	23.8	0
Ethanol	21.8	0

Table S2: Primer sequences (forward and reverse) of the investigated genes.

Gene	Sequence
Gapdh	5'-AACCTGCCAAGTATGATGAC-3'<br>5'-GGAGTTGCTGTTGAAGTCA-3'
MyoD	5'-GCTCTGATGGCATGATGG-3'<br>5'-CACTCTTCCCTGGTCTGG-3'
Myog	5'-TGAATGCAACTCCCACAG-3'<br>5'-CTGGCTTCTGCTGATATTGA-3'
Myh1	5'-GCGAGCAAATGATCTCCT-3'<br>5'-CTTCTTGTTAGACATGATCTGGTA-3'

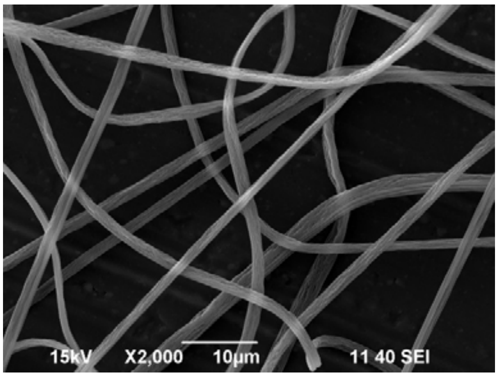

Figure S1: SEM image of the fibers produced by electrospinning the DMSO-PECA solution at 10.0% v/v in acetone.

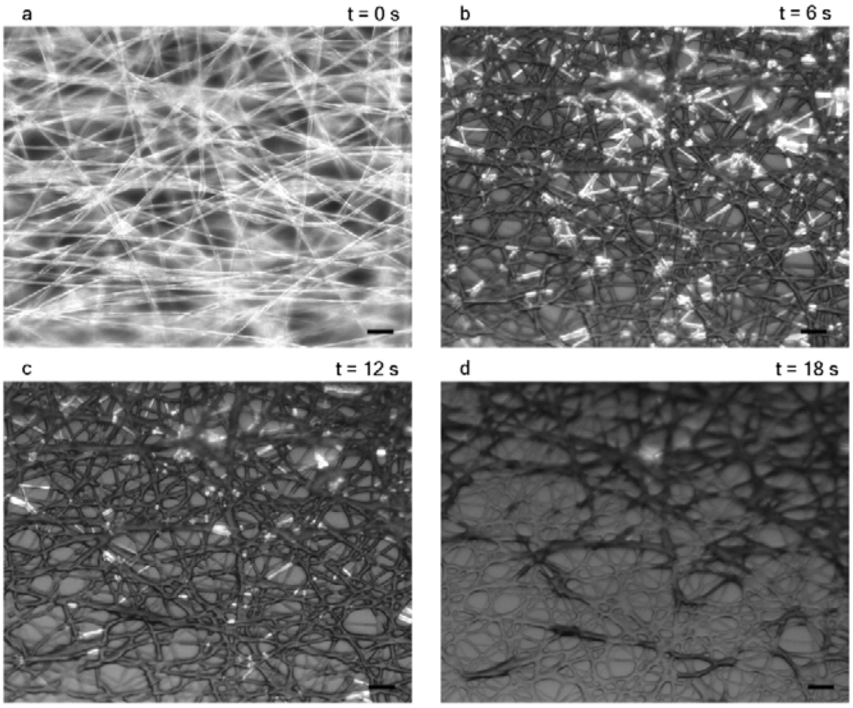

Figure S2: (a-d) Time sequence for the dynamics of formation of the textured PECA coatings. First, (a) the glass substrate, coated with a mat of PECA electrospun fibers, was placed in contact with the hot plate at 150 °C; the brightness of the fibers is an indication of the three-dimensional

(3D) organisation of the fibrous web and the presence of air. Immediately after contacting the hot surface (b-c) the adhesion of the fibers with the glass substrate increased and the thickness of the fibrous network decreased; only few bright regions were observed. Eventually, (c) a flat microstructured coating was created. Scale bar: 300 μm.

The Raman spectra of the fibrous mat before (black curve) and after (blue curve) the thermal treatment were shown for wavenumber ranges of 1200-2500 cm^{-1} (Figure S3a) and 2700-3200 cm^{-1} (Figure S3b): the $\delta(CH_3)$ deformation band at 1274 cm^{-1}, the ν(CH) stretching mode at 1450 cm^{-1}, the ester C=O group [ν(CO)] at 1744 cm^{-1}, the ν(CN) stretch of the nitrile (C≡N) groups at 2248 cm^{-1}, the ν(CH) stretching band at 2944 and 2974 cm^{-1} [1].

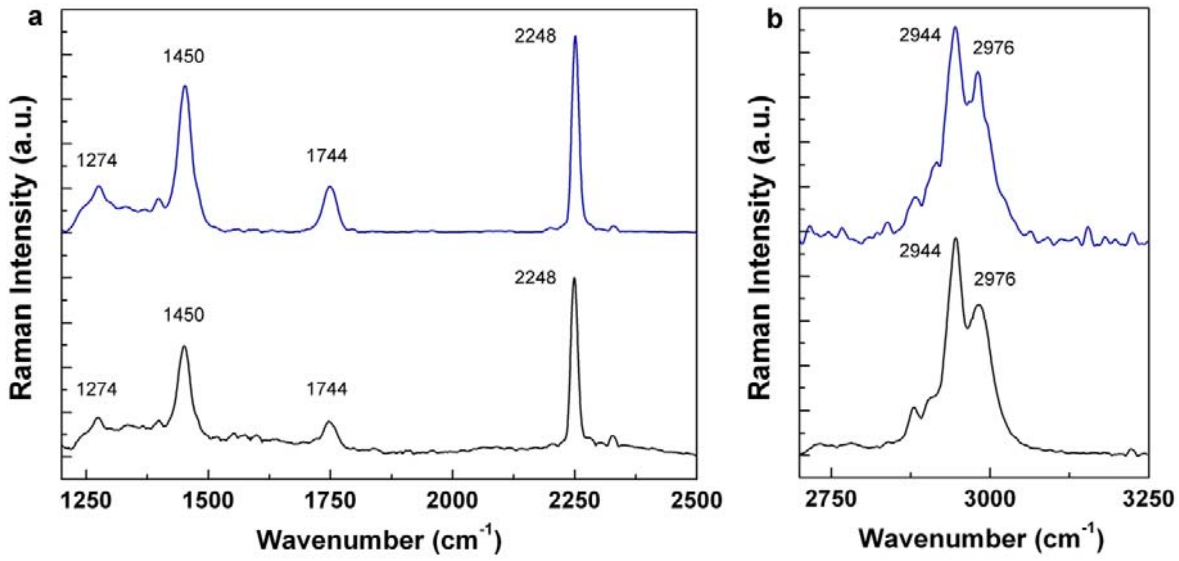

Figure S3: (a) and (b) Raman spectra of the PECA fibers before (black curve) and after (blue curve) the thermal treatment.

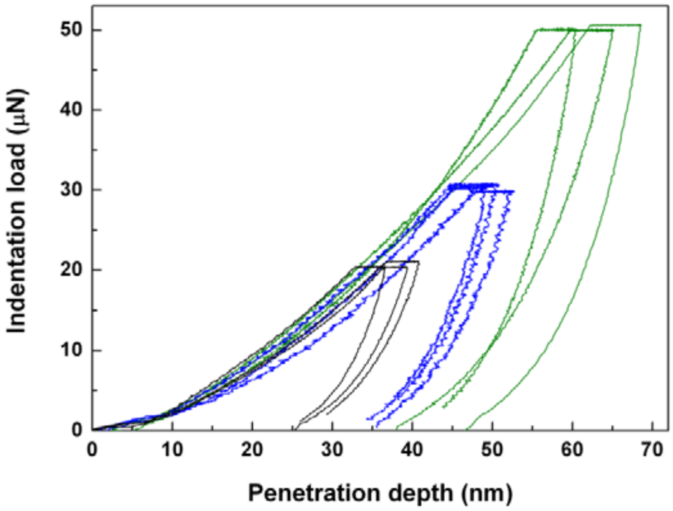

Figure S4: Selection of typical load-depth curves for the measurement of hardness of the studied PECA coatings at three maximum loads: 20 µN (black lines), 30 µN (blue lines) and 50 µN (green lines).

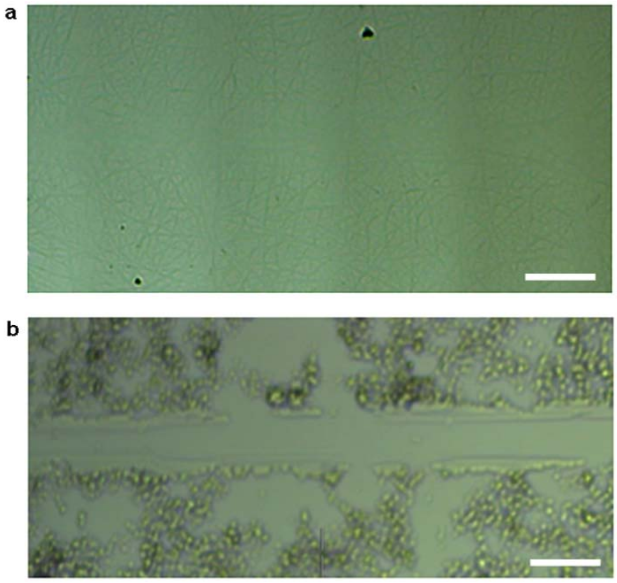

Figure S5: Images acquired by optical microscopy of the scratch produced on (a) the PECA coating and (b) the commercial dry lubricant Teflub. The scratch is difficult to see for the PECA coating, whereas it is clearly visible for the Teflub. Scale bar = 50 µm.

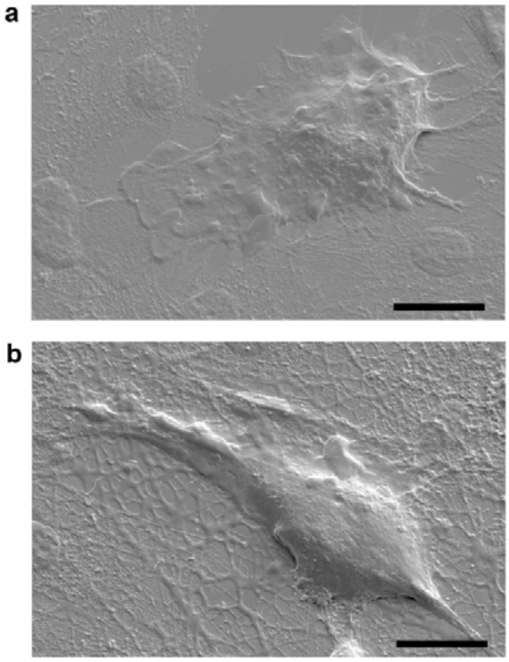

Figure S6: SEM images show that myoblasts well adhere and spread on (a) the glass substrate and on (b) the thermally treated PECA fibers. Scale bar = 20 µm.

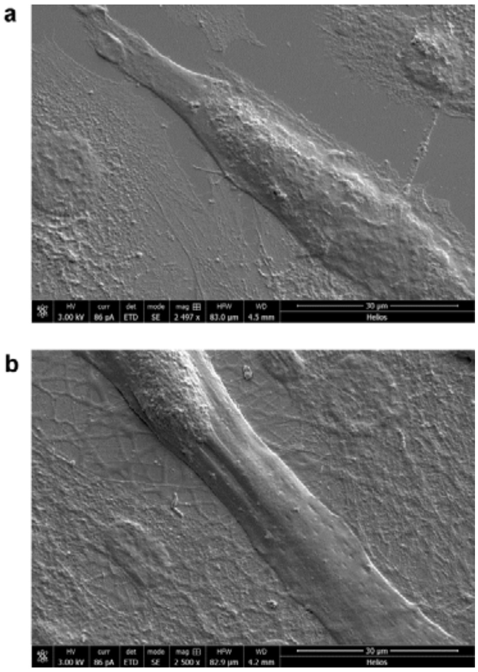

Figure S7: SEM images of C2C12 myoblasts after 72 h of differentiation on (a) glass and (b) PECA substrate.

References

1. Edwards, H. G. M. & Day, J. S. Fourier transform Raman spectroscopic studies of the curing of cyanoacrylate glue. *J. Raman Spectrosc*. **35**, 555-560 (2004).
